# hsa-miR-100-5p, an overexpressed miRNA in human ovarian endometriotic stromal cells, promotes invasion through attenuation of SMARCD1 expression

**DOI:** 10.1186/s12958-020-00590-3

**Published:** 2020-04-16

**Authors:** Kanetoshi Takebayashi, Kaei Nasu, Mamiko Okamoto, Yoko Aoyagi, Tomoko Hirakawa, Hisashi Narahara

**Affiliations:** 1 Department of Obstetrics and Gynecology, Faculty of Medicine, Oita University, Idaigaoka 1-1, Hasama-machi, Yufu-shi, Oita, 879-5593 Japan; 2 Division of Obstetrics and Gynecology, Support System for Community Medicine, Faculty of Medicine, Oita University, Oita, Japan

**Keywords:** Endometriosis, Invasion, Hsa-miR-100-5p, SMARCD1, Matrix metallopeptidase 1

## Abstract

**Background:**

A number of microRNAs are aberrantly expressed in endometriosis and are involved in its pathogenesis. Our previous study demonstrated that has-miR-100-5p expression is enhanced in human endometriotic cyst stromal cells (ECSCs). The present study aimed to elucidate the roles of has-miR-100-5p in the pathogenesis of endometriosis.

**Methods:**

Normal endometrial stromal cells (NESCs) were isolated from normal eutopic endometrium without endometriosis. Using hsa-miR-100-5p-transfected NESCs, we evaluated the effect of hsa-miR-100-5p on the invasiveness of these cells by Transwell invasion assay and in-vitro wound repair assay. We also investigated the downstream signal pathways of hsa-miR-100-5p by microarray analysis and Ingenuity pathways analysis.

**Results:**

hsa-miR-100-5p transfection enhanced the invasion and motility of NESCs. After hsa-miR-100-5p transfection, mRNA expression of SWItch/sucrose non-fermentable-related matrix-associated actin-dependent regulator of chromatin subfamily D member 1 (SMARCD1) was significantly attenuated. Whereas, the expression of matrix metallopeptidase 1 (MMP1) mRNA and active MMP1 protein levels was upregulated.

**Conclusion:**

We found that SMARCD1/MMP-1 is a downstream pathway of hsa-miR-100-5p. hsa-miR-100-5p transfection enhanced the motility of NESCs by inhibiting SMARCD1 expression and MMP1 activation. These findings suggest that enhanced hsa-miR-100-5p expression in endometriosis is involved in promoting the acquisition of endometriosis-specific characteristics during endometriosis development. Our present findings on the roles of hsa-miR-100-5p may thus contribute to understand the epigenetic mechanisms involved in the pathogenesis of endometriosis.

## Background

Endometriosis belongs to estrogen-dependent benign tumors and occurs in 6–10% of the women of reproductive age [1]. The microscopic features of endometriotic tissues resemble those of proliferative-phase endometrial tissues [1]; however, molecular studies have revealed a number of differences at the epigenetic, genetic, transcriptional, and posttranscriptional levels [2–5].

To understand the mechanism(s) responsible for the pathogenesis of endometriosis, we have previously investigated microRNA (miRNA) expression levels in endometriosis [4–7]. Our previous microarray study detected a repertoire of aberrantly expressed miRNAs in endometriosis [4]. Of these aberrantly expressed miRNAs, we demonstrated that upregulation of hsa-miR-210 [5] and downregulation of hsa-miR-196b [4] and hsa-miR-503 [6] contribute to the pathogenesis of endometriosis. Hsa-miR-210 induced the cell proliferation and vascular endothelial cell growth factor (VEGF) production of human normal endometrial stromal cells (NESCs) and inhibited apoptosis of these cells [5]. Whereas, hsa-miR-196b induced the apoptosis of human endometriotic cyst stromal cells (ECSCs) and inhibited the proliferation of these cells [4]. hsa-miR-503 also induced the cell-cycle arrest at G0/G1 phase and apoptosis and inhibited the cell proliferation, VEGF production, and contractility of ECSCs [6].

SWItch/sucrose non-fermentable (SWI/SNF)-related matrix-associatedactin-dependent regulator of chromatin subfamily D member 1 (SMARCD1) belongs to the SWI/SNF chromatin remodeling complex family of proteins which regulate the target gene transcription by altering the local chromatin structure around those genes [8, 9]. SMARCD1 is often involved in somatic rearrangement in tumorigenesis [10]. The chromatin remodeling activity of SMARCD1 is essential for tumor suppression [11, 12]. We speculated that SMARCD1 supression may induce tumorigenesis in endometriosis.

Matrix metallopeptidase 1 (MMP1) is a key enzyme that promotes the breakdown of extracellular matrix during physiological and pathological processes such as embryonic development, reproduction, and tissue remodeling, as well as tumor invasion and metastasis. MMP-1 is the most ubiquitously expressed interstitial collagenase that cleaves the interstitial collagen, types I, II, and III [13]. MMP1 is overexpressed in endometriotic tissues, suggesting its involvement in the pathogenesis of endometriosis [14].

In the present study, we evaluated the role of hsa-miR-100-5p, a miRNA that is upregulated in ECSCs, regarding the pathogenesis of endometriosis [4]. Using hsa-miR-100-5p-transfected NESCs, we assessed the effect of hsa-miR-100-5p on the invasiveness of these cells and the expression of SMARCD1 and MMP1, which are downstream targets of hsa-miR-100-5p, in these cells.

## Methods

### Human NESC and ECSC isolation procedures and cell culture conditions

Normal endometrial tissues were collected at the time of hysterectomies from patients with subserous or intramural leiomyoma who had regular menstrual cycle and had no evidence of endometriosis (*n* = 13, age 31**–**53 yrs.), as described previously [15]. Whereas, ovarian endometrioma tissues were obtained at the time of surgical treatment from patients with regular menstrual cycles (*n* = 6, age 22**–**42 yrs.), as described before [6, 7, 15]. None of the patients had received the hormonal treatments for at least 2 years prior to the surgery. Pathological examination and/or menstrual records confirmed that all the specimens were in the mid-to-late proliferative phases. This study was approved by the Institutional Review Board (IRB) of the Faculty of Medicine, Oita University (registration number: P-16-01), and written informed consent was obtained from all the patients.

ECSCs and NESCs were isolated from ovarian endometrioma and normal endometrial tissues, respectively, by enzymatic digestion, and cultured in Dulbecco’s modified Eagle’s medium (DMEM) supplemented with 100 IU/ml of penicillin (Gibco-BRL, Gaithersburg, MD, USA), 50 mg/ml of streptomycin (Gibco-BRL), and 10% charcoal-strippedheat-inactivated fetal bovine serum (FBS) (Gibco-BRL) at 37 °C in 5% CO_2_ in air, as described previously [6, 7, 15]. This culture condition is free of ovarian steroid hormones. Each experiment was performed in triplicate and was repeated at least three times with cells isolated from separate patients.

### Quantitative reverse transcription-polymerase chain reaction (RT-PCR) for hsa-miR-100-5p

In our previous study, using a miRNA microarray technique, we demonstrated that hsa-miR-100-5p was upregulated in ECSCs [4]. For the validation of the microarray data, we performed quantitative RT-PCR with NESCs (*n* = 6) and ECSCs (*n* = 6) as described previously [4–6]. hsa-miR-100-5p-specific (Assay ID: 000437, Applied Biosystems, Carlsbad, CA, USA) or endogenous control (RNU44)-specific (Assay ID: 001094, Applied Biosystems) reverse primers were used. The expression levels of hsa-miR-100-5p were normalized to those of RNU44, calculated by the ΔΔCT method, and were presented as the relative expression in ECSCs compared to that in NESCs.

### Transfection of miRNA precursors and small interfering RNAs (siRNAs)

Precursor hsa-miR-100-5p (pre-miR miRNA precursor-hsa-miR-100-5p, Ambion, Austin, TX, USA), negative control precursor miRNA (pre-miR miRNA precursor-negative control #1, Ambion), SMARCD1 silener pre-designed siRNA (AM16708, Ambion) or Silencer® select negative control #1 siRNA (Ambion) were transfected into NESCs using Lipofectamine RNAiMAX (Invitrogen, Carlsbad, CA, USA) and the reverse transfection method, as described before [4–6].

### Gene expression microarray

Forty-eight hours after transfection, total RNA was extracted from cultured NESCs transfected with precursor hsa-miR-100-5p (*n* = 4) and NESCs transfected with negative control precursor miRNA (*n* = 4) using an RNeasy Mini kit (Qiagen, Valencia, CA, USA) and subjected to gene expression microarray analyses with a commercially available human mRNA microarray (G4851A, SurePrint G3 Human Gene Expression Microarray 8x60K v2, Agilent Technologies, Santa Clara, CA, USA), as described previously [5]. To identify the upregulated and downregulated genes, the Z-scores and ratios (non-log scaled fold-change) from the normalized signal intensities of each probe were calculated to compare between NESCs transfected with precursor hsa-miR-100-5p and NESCs transfected with negative control precursor miRNA [5]. We established the following criteria for the regulated genes: at least 3 out of 4 samples has Z-score ≥ 2.0 and ratio ≥ 2.0-fold for upregulated genes, and Z-score ≤ − 2.0 and ratio ≤ 0.5 for downregulated genes. All the gene expression microarray data are available at the Gene Expression Omnibus through the NCBI under Accession No. GSE139954 (https://www.ncbi.nlm.nih.gov/geo/query/acc.cgi?acc=GSE139954).

### miRNA target prediction and pathways analysis

To elucidate the downstream target genes and signal pathways of hsa-miR-100-5p, datasets representing the genes with an altered expression profile derived from the microarray analyses were analyzed by the Ingenuity pathways analysis (IPA) software (Ingenuity Systems, Redwood City, CA, USA) with the IPA knowledgebase (IPA Summer Release 2015). Thereafter, predicted targets of hsa-miR-100-5p were confirmed by online public databases including miRDB (http://mirdb.org/miRDB/), TargetScanHuman (http://www.targetscan.org/, Release 7.0), PicTar (http://pictar.mdc-berlin.de/), and microRNA.org (http://www.microrna.org/microrna/getGeneForm.do).

### Transwell invasion assay

The invasive properties of hsa-miR-100-5p-transfected NESCs were evaluated by Transwell invasion assay, as described previously [16, 17]. NESCs after miRNA transfection (2 × 10^5^ cells) were cultured in DMEM supplemented with 10% charcoal-strippedheat-inactivated FBS on the growth factor-reducedMatrigel-coated Transwell inserts with 8-μm pores (Corning Inc., New York, NY, USA). After 48 h, the membranes were fixed with 100% methanol, and the number of cells appearing on the undersurface of the polycarbonate membranes after Giemsa staining was scored visually at × 200 magnification using a light microscope.

The data from triplicate samples were calculated and presented as the percent values obtained for the NESCs transfected with precursor hsa-miR-100-5p relative to those transfected with the negative control precursor miRNA.

### In vitro wound repair assay

Cell motility was also determined by an in vitro wound repair assay, as described previously [16, 17]. NESCs grown to confluence in 6-well plates (Corning Inc.) were challenged overnight with serum-free medium and then transfected with the miRNA precursor. The monolayer was wounded using a cell scraper and the plates were incubated in DMEM plus 0.1% BSA for 48 h. The cells were then fixed with 3% paraformaldehyde and stained with Giemsa solution. Areas with lesions were photographed, and wound repair was assessed by calculating the repaired area in square micrometers between the lesion edges at 0 h and 48 h using the public domain software Image J 1.44 developed at the U.S. National Institutes of Health (Bethesda, MD, USA).

The data from triplicate samples were calculated and presented as the percent values obtained for the NESCs transfected with precursor hsa-miR-100-5p relative to those transfected with the negative control precursor miRNA.

### RT-PCR for mRNA expression

**Fig. 1:**
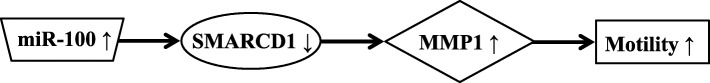
Downstream signaling pathway of hsa-miR-100-5p in NESCs. A gene expression microarray and pathway analyses of hsa-miR-100-5p-transfected NESCs revealed that hsa-miR-100-5p upregulated the motility of NESCs by direct inhibition of SMARCD1 expression followed by MMP1 activation. SMARCD1, SWItch/sucrose non-fermentable-related matrix-associated actin-dependent regulator of chromatin subfamily D member 1; MMP1, matrix metallopeptidase 1; NESCs, normal endometrial stromal cells

The effects of hsa-miR-100-5p on the expression levels of possible downstream target genes were evaluated in ECSCs by quantitative RT-PCR, as described [4–6]. *SMARCD1* and *MMP1* were selected as candidate genes because *SMARCD1* was confirmed to be the predicted target of hsa-miR-100-5p in the online public database, TargetScanHuman (http://www.targetscan.org/, Release 7.2). *MMP1* is known to be the downstream target of *SMARCD1* [18] and promotes cell motility (Fig. 1).

In brief, 48 h after miRNA transfection, total RNA from miRNA-transfected NESCs was extracted as described above and subjected to quantitative RT-PCR with the following specific primers (all from Applied Biosystems): *SMARCD1* (Assay ID: Hs00161980_m1), *MMP1* (Assay ID: Hs00899658_m1), or glyceraldehyde 3-phosphate dehydrogenase (*GAPDH*) (Assay ID: Hs02758991_g1). The expression levels of candidate mRNAs relative to those of *GAPDH* mRNA were calculated using a calibration curve. The data were calculated from triplicate samples and are presented as percent values obtained for NESCs after hsa-miR-100-5p transfection relative to those transfected with the negative control precursor miRNA.

### ELISA for active MMP1

Culture media of miRNA-transfected NESCs were collected 48 h after miRNA transfection and subjected to Human Active MMP-1 Fluorescent Assay (F1M00, R&D Systems, Minneapolis, MN, USA), according to the manufacturer’s instructions. The data from triplicate samples were calculated and presented as the percent values obtained for NESCs transfected with precursor hsa-miR-100-5p relative to those transfected with the negative control precursor miRNA.

### Statistical analysis

All data were obtained from triplicate samples and are presented as percent values relative to the corresponding controls in the form of mean ± SD. Data were appropriately analyzed by the Student’s *t*-test using the Statistical Package for Social Science software (IBM SPSS statistics 24; IBM, Armonk, NY, USA). *P*-values < 0.05 were considered statistically significant.

## Results

### Expression of hsa-miR-100-5p

**Fig. 2:**
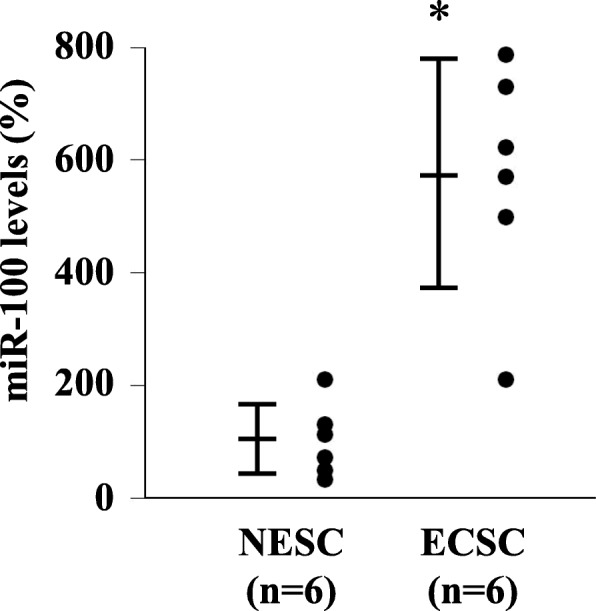
hsa-miR-100-5p expression in NESCs and ECSCs. The relative hsa-miR-100-5p levels in ECSCs (*n* = 6) were significantly higher than those in the NESCs (*n* = 6). *p < 0.0005 vs. NESCs (Student’s *t*-test). Data are shown as the mean ± SD. ECSCs, endometriotic cyst stromal cells; NESCs, normal endometrial stromal cells

To validate the miRNA microarray data [4], we evaluated the hsa-miR-100-5p expression levels in NESCs and ECSCs using quantitative RT-PCR. As shown in Fig. 2, the relative hsa-miR-100-5p levels in the ECSCs were significantly higher than those in the NESCs (*p* < 0.0005). Thus, the results of quantitative RT-PCR for hsa-miR-100-5p expression were consistent with our previous miRNA microarray data [4]. Age of the patients did not affect the expression of hsa-miR-100-5p (data not shown).

**Fig. 3:**
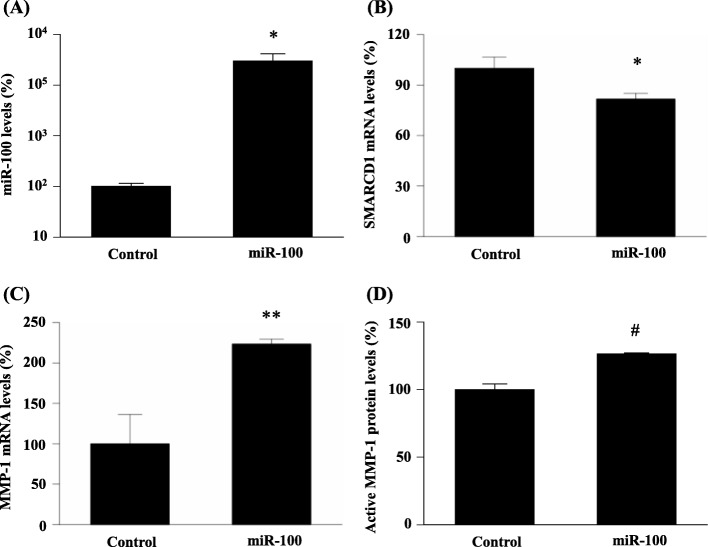
Effects of hsa-miR-100-5p transfection on the downstream target molecule expression in NESCs. (**a**) hsa-miR-100-5p expression after precursor miRNA transfection. Note that the vertical axis is expressed as a logarithmic scale. (**b**) *SMARCD1* mRNA expression. (**c**) *MMP1* mRNA expression. (**d**) Active MMP1 protein expression. *p < 0.05, **p < 0.005, #*p* < 0.0005 vs. controls (Student’s *t*-test). MMP1, matrix metallopeptidase 1; NESCs, normal endometrial stromal cells; SMARCD1, SWItch/sucrose non-fermentable-related matrix-associated actin-dependent regulator of chromatin subfamily D member 1

As shown in Fig. 3a, mature hsa-miR-100-5p expression in NESCs was significantly induced by hsa-miR-100-5p precursor transfection (*p* < 0.05). We thus considered this experimental model as appropriate for hsa-miR-100-5p functional analyses.

### Identification of hsa-miR-100-5p-regulated genes and predicted pathways in NESCs

As shown in Table 1, gene expression microarray analyses detected 33 upregulated and 27 downregulated mRNAs using the criteria described above. Using the online public databases, we focused on SMARCD1 involved in the pathogenesis of endometriosis. The IPA software then identified MMP1 as a downstream target of SMARCD1 (Fig. 1). Regarding the known function of MMP1, we evaluated the cell motility of NESCs using the following experiments.

**Table 1:** List of mRNAs aberrantly expressed in miR-100-5p-transfected NESCs

Gene family	Gene symbol	Control signal	miR-100 precursor signal	Z-score	Ratio
(A) Upregulated mRNAs					
Cytokine	*CCL2*	312.22	1041.65	6.87	4.32
	*IL11*	1034.43	2782.35	5.98	3.11
	*LIF*	128.77	261.89	3.80	2.46
	*RP2*	838.00	1737.49	4.13	2.17
Growth factor	*BMP2*	817.37	461.26	−4.41	0.45
Peptidase	*MMP1*	49,499.07	74,041.18	7.47	2.40
	*CPXM1*	70.96	120.43	2.27	2.40
Enzyme	*ASPH*	1259.03	3722.52	6.99	3.81
	*CYBRD1*	1067.64	1867.57.94	4.18	2.08
	*MTAP*	440.73	714.19	3.88	2.18
Transcription regulator	*UBE2V1*	2487.25	6026.87	5.85	2.87
	*SCML1*	48.29	98.99	3.02	2.53
	*BATF3*	147.71	291.50	3.05	2.02
Transmembrane receptor	*ITGA6*	1383.58	2680.82	4.48	2.21
	*PVRL2*	6699.21	13,211.87	4.72	2.04
Transporter	*ABCA1*	134.70	270.47	3.29	2.12
	*BCAP29*	1166.81	1994.48	4.09	2.03
Other	*TUBB2B*	41.72	123.42	4.64	3.54
	*PKIA*	192.01	593.66	5.84	3.20
	*PALM3*	260.83	736.98	5.72	2.99
	*CEND1*	196.72	412.79	4.30	2.80
	*EMC10*	4713.79	9455.68	5.88	2.76
	*ERLIN2*	225.65	451.23	3.82	2.41
	*TFPI2*	752.91	1839.06	4.64	2.35
	*RDX*	469.66	816.51	4.11	2.27
	*FAM131B*	47.52	91.03	2.61	2.24
	*SWAP70*	440.11	805.17	4.01	2.18
	*LOC728392*	1643.31	3433.27	4.56	2.15
	*STBD1*	104.93	162.95	3.20	2.15
	*SNRPC*	10,269.42	17,744.24	4.89	2.12
Other	*CIDEC*	488.37	852.48	3.68	2.05
	*ANGPTL4*	1403.18	2829.40	4.28	2.01
Null	*LOC102723946*	38.79	110.21	3.88	3.33
(B) Downregulated mRNAs					
Enzyme	*HSD17B2*	286.50	128.94	−5.76	0.26
	*PLCH1*	190.28	88.66	−4.24	0.38
	*INMT*	1404.19	713.49	−5.10	0.40
Ion channel	*KCNN2*	118.10	32.10	−4.34	0.24
	*KCTD4*	151.44	53.14	−5.41	0.28
	*KCTD10*	1074.65	563.34	−4.14	0.47
Kinase	*FGFR3*	239.75	121.71	−3.31	0.49
Peptidase	*ADAMTS5*	3143.19	1023.39	−6.74	0.33
	*ADAM19*	239.21	117.14	−3.26	0.47
Phosphatase	*LPPR4*	184.80	73.50	−4.33	0.34
Transcription regulator	*SMARCD1*	2210.80	932.50	−5.34	0.43
	*BAZ2A*	288.00	135.67	−3.32	0.47
Transporter	*ATP6AP1*	9337.60	3682.26	−6.29	0.40
Other	*EPDR1*	3173.94	780.35	−8.19	0.27
	*MPZL3*	125.86	59.79	−3.82	0.43
	*ZBED2*	161.28	71.97	−3.74	0.43
	*SUDS3*	568.38	289.31	−4.34	0.46
	*CGA*	112.70	61.15	−3.01	0.46
	*TMEM30A*	3229.37	1492.02	−4.80	0.47
	*DGCR2*	4328.05	2074.04	−4.65	0.47
	*CTDSPL*	748.62	362.73	−4.11	0.48
	*DEPTOR*	398.87	187.27	−3.37	0.48
	*DNAJC11*	1560.93	839.74	−3.46	0.49
	*CLDN11*	1351.65	687.71	−3.46	0.49
Null	*SLC16A14*	144.45	54.61	−3.92	0.35
	*PROSER2-AS1*	35.66	16.12	−2.11	0.42
	*AREG*	120.26	61.28	−3.53	0.43

*ABCA1* ATP-binding cassette, sub-family A, member 1; *ADAM19* ADAM metallopeptidase domain 19; *ADAMTS5* ADAM metallopeptidase with thrombospondin type 1 motif, 5; *ANGPTL4* angiopoietin-like 4 transcript variant 1; *AREG* amphiregulin; *ASPH* aspartate beta-hydroxylase, transcript variant 3; *ATP6AP1* ATPase, H+ transporting, lysosomal accessory protein 1; *BATF3* basic leucine zipper transcription factor, ATF-like 3; *BAZ2A* bromodomain adjacent to zinc finger domain, 2A; *BCAP29* B-cell receptor-associated protein 29, transcript variant 2; *BMP2* bone morphogenetic protein 2; *CCL2* chemokine ligand 2; *CEND1* cell cycle exit and neuronal differentiation 1; *CGA* glycoprotein hormones, alpha polypeptide, transcript variant 2; *CIDEC* cell death-inducing DFFA-like effector c, transcript variant 3; *CLDN11* claudin 11, transcript variant 1; *CPXM1* carboxypeptidase X, member 1, transcript variant 1; *CTDSPL* CTD (carboxy-terminal domain, RNA polymerase II, polypeptide A) small phosphatase-like, transcript variant 1; *CYBRD1* cytochrome b reductase 1, transcript variant 1; *DEPTOR* DEP domain containing MTOR-interacting protein, transcript variant 1; *DGCR2* DiGeorge syndrome critical region gene 2, transcript variant 1; *DNAJC11* DnaJ (Hsp40) homolog, subfamily C, member 11; *EMC10* ER membrane protein complex subunit 10, transcript variant 1; *EPDR1* ependymin related 1, transcript variant 1; *ERLIN2* ER lipid raft associated 2, transcript variant 1; *FAM131B* family with sequence similarity 131, member B, transcript variant a; *FGFR3* fibroblast growth factor receptor 3, transcript variant 1; *HSD17B2* hydroxysteroid (17-beta) dehydrogenase 2; *IL11* interleukin 11; *INMT* indolethylamine N-methyltransferase, transcript variant 2; *ITGA6* integrin, alpha 6, transcript variant 2; *KCNN2* potassium channel, calcium activated intermediate/small conductance subfamily N alpha, member 2, transcript variant 1; *KCTD10* potassium channel tetramerization domain containing 10; *KCTD4* potassium channel tetramerization domain containing 4; *LIF* leukemia inhibitory factor, transcript variant 1; LOC102723946, Zinc finger protein 695; LOC728392, uncharacterized LOC728392; *LPPR4* lipid phosphate phosphatase-related protein type 4, transcript variant 1; *MMP1* matrix metallopeptidase 1, transcript variant 1; *MPZL3* myelin protein zero-like 3, transcript variant 1: *MTAP* methylthioadenosine phosphorylase; *PALM3* paralemmin 3; *PKIA* protein kinase (cAMP-dependent, catalytic) inhibitor alpha, transcript variant 1; *PLCH1* phospholipase C, eta 1, transcript variant 2; *PROSER2-AS1* PROSER2 antisense RNA 1; *PVRL2* poliovirus receptor-related 2, transcript variant delta; *RDX* radixin, transcript variant 3; *RP2* retinitis pigmentosa 2; *SCML1* sex comb on midleg-like 1, transcript variant 1; *SLC16A14* solute carrier family 16, member 14; *SMARCD1* SWI/SNF related, matrix associated, actin dependent regulator of chromatin, subfamily d, member 1, transcript variant 2; *SNRPC* small nuclear ribonucleoprotein polypeptide C, transcript variant 1; *STBD1* starch binding domain 1; *SUDS3* suppressor of defective silencing 3 homolog; *SWAP70* SWAP switching B-cell complex 70 kDa subunit, transcript variant 1; *TFPI2* tissue factor pathway inhibitor 2, transcript variant 1; *TMEM30A* transmembrane protein 30A, transcript variant 1; *TUBB2B* tubulin, beta 2B class IIb; *UBE2V1* ubiquitin-conjugating enzyme E2 variant 1, transcript variant 4; *ZBED2* zinc finger, BED-type containing 2

### Modulation of downstream target molecule expression by hsa-miR-100-5p transfection

To investigate the underlying mechanisms of hsa-miR-100-5p functions, we investigated the expression levels of SMARCD1 and MMP1. As shown in Fig. 3b, *SMARCD1* mRNA expression was significantly attenuated by hsa-miR-100-5p transfection (*p* < 0.05). In contrast, as shown in Fig. 3c and d, the expression levels of *MMP1* mRNA, and active MMP1 protein were upregulated by hsa-miR-100-5p transfection (*p* < 0.005 and *p* < 0.0005, respectively).

### Modulation of MMP1 expression by SMARCD1 siRNA transfection

**Fig. 4:**
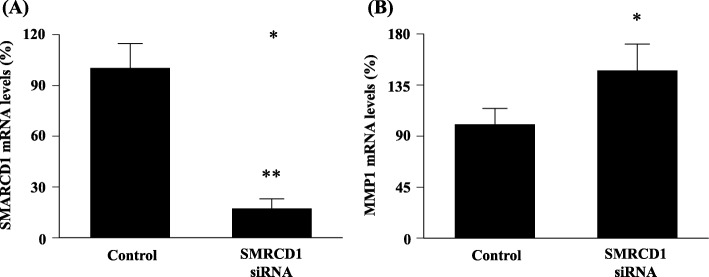
Effects of SMARCD1 siRNA transfection on the MMP1 mRNA expression in NESCs. (**a**) *SMARCD1* mRNA expression after SMARCD1 siRNA transfection. (**b**) *MMP1* mRNA expression. *p < 0.05, **p < 0.005 vs. controls (Student’s *t*-test). MMP1, matrix metallopeptidase 1; NESCs, normal endometrial stromal cells; SMARCD1, SWItch/sucrose non-fermentable-related matrix-associated actin-dependent regulator of chromatin subfamily D member 1

To confirm that the MMP1 expression is regulated by SMARCD1, we investigated the expression levels of MMP1 mRNA after SMARCD1 siRNA transfection. As shown in Fig. 4a, *SMARCD1* mRNA expression was significantly suppressed by SMARCD1 siRNA transfection (*p* < 0.005). As shown in Fig. 4b, the expression levels of *MMP1* mRNA was significantly upregulated by SMARCD1 siRNA transfection (*p* < 0.05).

### Cell motility

**Fig. 5:**
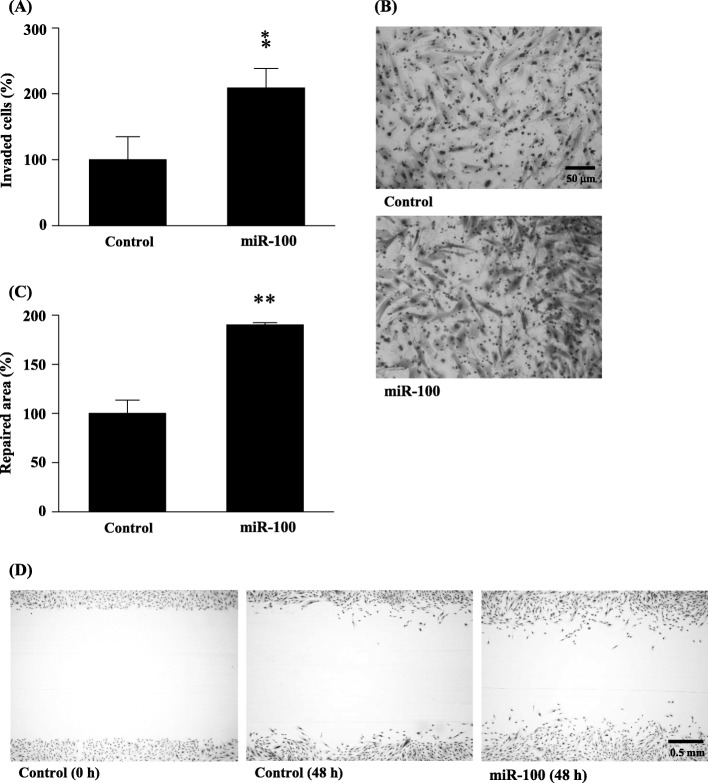
Effects of hsa-miR-100-5p transfection on the motility of NESCs. (**a**) Results of transwell invasion assay. (**b**) Representative photographs of transwell invasion assay. (**c**) Results of in-vitro wound repair assay. (**d**) Representative photographs of in vitro wound repair assay. *p < 0.05, **p < 0.0005 vs. controls (Student’s *t*-test). NESCs, normal endometrial stromal cells

As shown in Fig. 5a and b, the transwell invasion assay revealed that the number of invaded cells was significantly increased by hsa-miR-100-5p transfection (*p* < 0.05).

We also investigated the effects of hsa-miR-100-5p on the motility of NESCs by an in vitro wound healing assay. As shown in Fig. 5c and d, the repaired area was significantly increased by hsa-miR-100-5p transfection (*p* < 0.0005).

## Discussion

To understand the role of hsa-miR-100-5p, which is upregulated in ECSCs, in the pathogenesis of endometriosis, we evaluated its expression in both ECSCs and NESCs. We also evaluated the hsa-miR-100-5p-mediated effects on the cellular functions of NESCs and sought to determine the underlying mechanisms of hsa-miR-100-5p action in those cells. With the present study, we found the following: (1) Expression of hsa-miR-100-5p in ECSCs was upregulated compared to that in NESCs. (2) hsa-miR-100-5p transfection enhanced the motility of NESCs. (3) hsa-miR-100-5p promoted these cellular functions through downregulation of *SMARCD1* mRNA and induction of MMP1 expression. This suggests that hsa-miR-100-5p overexpression induces NESCs to acquire the highly motile characteristics of endometriosis and is involved in promoting the development and progression of this disease.

hsa-miR-100-5p can act as either a tumor suppressor gene or an oncogene, depending on the tumor type in different cancers [19, 20]. For example, hsa-miR-100-5p overexpression has been demonstrated in nasopharyngeal cancer [21], esophageal squamous cell carcinoma [22], colon cancer [19, 23], and gastric cancer [24]. In these tumors, this miRNA contributes to tumor progression. In contrast, hsa-miR-100-5p expression is suppressed in epithelial ovarian cancer [25], endometrial cancer [26], bladder carcinoma [27], renal cell carcinoma [28], prostate cancer [29], breast carcinoma [30], hepatocellular carcinoma [31], and non-small cell lung cancer [32]. In these tumors, this miRNA behaves as a tumor suppressor.

The reported target genes of hsa-miR-100-5p include polo-like kinase 1 [21, 32], insulin-like growth factor (IGF) [33], IGF-1 receptor [34], mammallian target of rapamycin (mTOR) [34], fibroblast growth factor receptor 3 [35], ataxia telangiectasia mutated (ATM) [36], Argonaute 2 [37], isoprenylcysteine carboxyl methyltransferase (ICMT) [38], nuclear factor-κB3 [39], ras-related C3 botulinum toxin substrate 1 (Rac1) [38], and β-tubulin [40].

To our knowledge, there is no report which evaluated the expression and function of SMARCD1 in endometriosis. Whereas, overexpression of MMP1 is reported in endometriotic tissues [14], however, the roles of MMP1 regarding the pathogenesis of endometriosis has not been elucidated yet. MMP1 gene polymorphisms may also affect the motility of ECSCs [13]. In the present study, we demonstrated that transfection with hsa-miR-100-5p induced MMP1 expression in NESCs through downregulation of SMARCD1 and that MMP1 accelerated the migration of NESCs.

A limitation of the present study is that the experiments were performed only with the stromal cells of endometriosis and the eutopic endometrium of women without endometriosis. Due to difficulties in obtaining samples, the expression of hsa-miR-100-5p in the eutopic endometrium of women with endometriosis was not evaluated. Future study is necessary on this point.

## Conclusions

In summary, we confirmed that hsa-miR-100-5p expression is upregulated in ECSCs. By transfecting hsa-miR-100-5p into NESCs, we observed that SMARCD1/MMP-1 is the downstream pathway of hsa-miR-100-5p. Inhibition of SMARCD1 mRNA expression, followed by MMP1 activation, enhanced the motility of NESCs. These findings suggest that enhanced expression of hsa-miR-100-5p in endometriosis is involved a role in promoting the acquisition of endometriosis-specific characteristics during the development of endometriosis. Our present findings on the roles of hsa-miR-100-5p may thus contribute to understand the epigenetic mechanisms involved in the pathogenesis of endometriosis.

## Data Availability

All the gene expression microarray data are available at the Gene Expression Omnibus through the NCBI under Accession No. GSE139954 (https://www.ncbi.nlm.nih.gov/geo/query/acc.cgi?acc=GSE139954). Other data in this study are asvailable from the corresponding author.

## Abbreviations

ATM: Ataxia telangiectasia mutated
DMEM: Dulbecco's modified Eagle medium
ECSCs: Endometriotic cyst stromal cells
ELISA: Enzyme-linked immunosorbent assay
FBS: Fetal bovine serum
GAPDH: Glyceraldehyde 3-phosphate dehydrogenase
ICMT: Isoprenylcysteine carboxyl methyltransferase
IGF: Insulin-like growth factor
IPA: Ingenuity pathways analysis
IRB: Institutional Review Board
miRNA: microRNA
MMP1: Matrix metallopeptidase 1
mTOR: Mammallian target of rapamycin
NESCs: Normal endometrial stromal cells
Rac1: Ras-related C3 botulinum toxin substrate 1
RT-PCR: Reverse transcription-polymerase chain reaction
siRNA: Small interfering RNA
SMARCD1: SWI/SNF-related matrix-associatedactin-dependent regulator of chromatin subfamily D member 1
SWI/SNF: SWItch/sucrose non-fermentable
